# Cardiovascular Risk Factors in Transgender People after Gender-Affirming Hormone Therapy

**DOI:** 10.3390/jcm12196141

**Published:** 2023-09-23

**Authors:** Esteban Sánchez-Toscano, Jesús Domínguez-Riscart, Laura Larrán-Escandón, Isabel Mateo-Gavira, Manuel Aguilar-Diosdado

**Affiliations:** 1 Endocrinology and Nutrition Department, Puerta del Mar University Hospital, 11009 Cádiz, Spain; estebansanchez1994@gmail.com (E.S.-T.); laura.larran@hotmail.com (L.L.-E.); manuel.aguilar.sspa@juntadeandalucia.es (M.A.-D.); 2 Biomedical Research and Innovation Institute of Cádiz (INiBICA), 11009 Cádiz, Spain; jesus.dominguezriscart@gmail.com; 3 Pediatrics and Specific Areas Department, Puerta del Mar University Hospital, 11009 Cádiz, Spain; 4 School of Medicine, Cadiz University (UCA), 11003 Cádiz, Spain

**Keywords:** transgender, cardiovascular risk factors, metabolic comorbidities, cross-sex hormone therapy, gender-affirming hormones

## Abstract

Introduction: In the last decade, healthcare for the transgender population has increased considerably in many countries thanks to depathologization movements and the easier accessibility of medical assistance. The age at which they request to start gender-affirming hormones (GAHs) is increasingly younger. The cardiovascular risk associated with hormonal treatment is a novel research field, and the published studies are heterogeneous and inconclusive. Our objective is to determine the metabolic impact of GAHs in the transgender people treated in our Gender Identity Treatment Unit. Methods: We designed a pre–post study to analyze changes in anthropometric parameters (weight and body mass index), analytical determinations (fasting blood glucose, glycated hemoglobin, and lipoproteins), and blood pressure control in the transgender population treated with GAHs in Puerta del Mar University Hospital. These variables were collected before and one year after hormonal therapy. Results: A total of 227 transgender people were recruited between 2017 and 2020, 97 (40.09%) transwomen and 136 (59.91%) transmen. The average age at which GAHs began was 18 years. Weight, body mass index, and blood pressure increased significantly in both genders. Transmen showed a more atherogenic lipid profile, with a decrease in cholesterol LDL (*p* < 0.001) and an increase in triglycerides (*p* < 0.001). The risk of developing prediabetes or diabetes did not increase one year after treatment, although non-specific alterations in carbohydrate metabolism were detected, such as an increase in glycated hemoglobin in transmen (*p* = 0.040) and fasting blood glucose in transwomen (*p* = 0.008). No thromboembolic processes or cardiovascular events were reported during the first year of treatment. Conclusion: In our setting, transgender people developed changes in their metabolic profiles in the first year after hormonal treatment. Both transmen and transwomen showed early alterations in lipid and carbohydrate metabolism, slight elevations in blood pressure, and a tendency to gain weight. This makes lifestyle interventions necessary from the beginning of GAHs.

## 1. Introduction

Transgender is used to describe a person whose gender identity is different from their sex assigned at birth [1]. In the last decade, the care demand from transgender people has increased due to depathologization movements, greater public awareness, the easier accessibility of the healthcare service, and lesbian, gay, bisexual, transgender, and queer (LGBTQ) support groups. Consequently, the number of transgender people with hormone treatment is growing significantly [2].

Health care for transgender people in Spain is different depending on the Autonomous Community. In 2014, after the approval of the Law for Non-Discrimination based on Gender Identity and Recognition of Rights of Transgender People of Andalusia, a Gender Identity Treatment Unit was created in each province, made up of multidisciplinary teams (integrating gynecologists, urologists, otorhinolaryngologists, psychologists, pediatricians, plastic surgeons, and family doctors, with endocrinologists as coordinators).

Clinical practice in our province is realized according to the Integrated Care Process for Health Care for Transsexual People of the Ministry of Health and Families [3]. The treatments financed in our public health system are puberty blockers, gender-affirming hormones (GAHs), mastectomy, and hysterectomy for transmen and orchiectomy and feminizing genitoplasties in transwomen. Before starting hormonal therapy, the patient’s signature is necessary and, in the event that he is under 16 years of age, the signature of both parents (or legal guardian in his absence) is necessary. Gender reaffirmation surgery may be considered in patients older than 18 years who have been in treatment with GAHs for a minimum of 12 months with correct therapeutic adherence.

Traditionally, psychiatric evaluation was necessary to gain access to hormonal therapy, but nowadays, a favorable report from a mental health professional is not necessary to start GAHs [4]. In our hospital, we have two psychologists who accompany people throughout the transition process with cognitive behavioral therapy if they request it. In the event of the detection of any negative social factor that affects the patient’s health, the social work units are consulted.

Pubertal suppression with gonadotropin-releasing hormone analogs is proposed for transgender youth whose pubertal development is not complete in order to give more time for identity exploration [5]. The purpose of gender-affirming hormones is to accommodate the secondary sexual characteristics to the affirmed gender through the suppression of the endogenous sex hormone secretion determined by the gonadal sex and to maintain sex hormone levels within the normal range for the affirmed gender (clinical practice guidelines recommend testosterone for transmen and estrogens combined with anti-androgen for transwomen) [6].

The most common complications of estrogen treatment that have been described are hypertransaminasemia, dyslipidemia, and thromboembolic events [7]. The thrombotic risk seems to be due to procoagulant changes in the hemostatic system and the development of resistance to activated protein C; however, the studies that describe an increased risk of cardiovascular and thromboembolic morbidity and mortality in the transwomen who used ethinyl estradiol as estrogen are now obsolete for this indication [8]. Testosterone is associated with polycythemia, liver dysfunction, weight gain, altered lipid profile, higher blood pressure, and obstructive sleep apnea/hypopnea syndrome [9]. Several studies have been published concerning the metabolic effects of GAHS in both transgender men and women, although the results are often contradictory and inconclusive.

Recent studies suggest that there is an increase in body mass index in transgender people with GAHs; the studies report higher rates of obesity and earlier weight gain in transmen [10]. The results that describe changes in body composition are controversial, although it appears that GAHs are related to feminine body fat distribution and a lower waist–hip ratio in transwomen and to masculine body fat distribution with a lower hip circumference in transmen [11]. It is well established that excess body adiposity is associated with insulin resistance, decreased adiponectin levels, atherogenic dyslipidemia and secretion of pro-inflammatory cytokines, all of which entail an increased CVRF [12]. Likewise, insulin resistance has been described in cisgender men with hypogonadism and in cisgender women with polycystic ovary syndrome [13]; therefore, it would be expected in transmen who received testosterone and in transwoman who received anti-androgen drugs.

Both estrogen and testosterone treatment can alter the lipid profile, although the mechanisms behind these changes after GAHs remain unknown. A systematic review with low-quality evidence suggests that testosterone may increase LDL cholesterol and triglyceride levels and decrease HDL cholesterol levels in transmen, whereas oral estrogens may increase triglyceride levels in transwomen without changes in the rest of the parameters [14]. Similar results are obtained in adolescents who started GAHs after puberty suppression [15]. Current evidence shows that systolic and diastolic blood pressure (SBP/DBP) increased or remained stable in transmen, while it decreased or remained stable in transwoman [16].

However, the literature about CVRF effects in transgender people receiving the hormonal treatment recommended by clinical practice guidelines and current consensus documents is based mainly on observational and retrospective studios, with small sample sizes, short follow-up times, and heterogeneous populations. Our objective is to analyze changes in the metabolic profile of transgender people treated at our hospital.

## 2. Materials and Methods

### 2.1. Study Design and Study Population

We designed a retrospective before-and-after study to evaluate changes in the anthropometric parameters and CVRF in the transgender youth and adults cared for in the Gender Identity Treatment Unit of Hospital Puerta del Mar (Cádiz). The inclusion criteria were the following: transgender people over 14 years who started GAHs in our center between January 2017 and December 2020 and completed at least one year of treatment. The study was approved by the ethics committee of Puerta del Mar University Hospital (ethical approval code number PEIBA 0474-N-22).

In order to provide permission to use the data in this study, all the participants signed an informed consent form approved by the ethics committee. In the case of participants under 18 years old, signed consent was given by at least one of their parents (or their legal guardian in their absence). Patients who lost the follow-up or refused to sign the informed consent form were excluded from the study.

### 2.2. Treatment Protocol

The transmen received injections of testosterone cypionate monthly, starting with 100 mg and adjusting the dose every 3–4 months (the maximum dose was 200 mg every two weeks). The transwomen received the minimum dose of cyproterone acetate (usually 50 mg daily) necessary to suppress gonadotropins and oral 17-β-estradiol, starting with 1–2 mg per day; this was increased every 3–4 months (the maximum dose was 6 mg per day).

### 2.3. Evaluation of Variables Collected

The variables collected included the following: demographic data, anthropometric measurements, types of surgery performed, routine blood chemistry analyses, blood pressure level, and metabolic comorbidities (diabetes mellitus, arterial hypertension, and dyslipidemia). These variables were analyzed before and one year after gender affirming hormones.

Weight was measured in underwear without shoes using a MOBBA platform and MOBBA viewer V-201 (Barcelona, Spain). Height was measured to the nearest centimeter with a Harpenden stadiometer (Crymych, Pembs., UK). Blood pressure was determined with a Riester Minimus II Aneroid Sphygmomanometer (Jungingen, Germany) after five minutes of rest. The recorded value was the average of the second and third measurements, separated by one or two minutes. Plasma glucose and lipids were determined in venous blood using the Modular DPD biochemistry system (Roche Diagnostics, Basel, Switzerland). HbA_1c_ was determined in a Cobas Integra 700 analyzer (Roche Diagnostics, Basel, Switzerland), using an immunoturbidimetric method for whole anticoagulated venous blood. LDL cholesterol was calculated using the Friedewald formula.

Weight was classified according to BMI [17]: underweight (<18.5 kg/m^2^), normal weight (18.5–24.9 kg/m^2^), overweight class I (25–26.9 kg/m^2^), overweight class II (27–29.9 kg/m^2^), obesity class I (30–34.9 kg/m^2^), obesity class II (35–39.9 kg/m^2^), obesity class III (40–49.9 kg/m^2^), and obesity class IV (≥50 kg/m^2^).

Diagnosis of diabetes mellitus was performed according to the American Diabetic Association (ADA) criteria [18]: random plasma glucose ≥ 200 mg/dL (≥11.1 mmol/mol) in patients with classic symptoms of hyperglycemia; fasting blood glucose (FBG) ≥ 126 mg/dL (≥6.99 mmol/L); or glycated hemoglobin (HbA_1c_) ≥ 6.5% (48 mmol/L). The diagnostic criteria of prediabetes were an FBG of 100–125 mg/dL (5.6–6.9 mmol/L) or an HbA_1c_ of 5.7–6.4% (39–47 mmol/mol) [18].

Diagnosis of dyslipidemia was based on the ESC/EAS Guidelines for the management of dyslipidemias: total cholesterol ≥ 200 mg/dL (≥ 5.18 mmol/L); LDL cholesterol ≥ 160 mg/dL (≥3.37 mmol/L); or triglycerides ≥ 150 mg/dL (≥1.8 mmol/L); arterial hypertension was defined as levels of SBP ≥ 140 mmHg and/or DBP ≥ 90 mmHg [19].

### 2.4. Statistical Analyses

Statistical analyses were performed using the SPSS program (version 23.0 for Windows). The descriptive analyses included frequencies and percentages for the qualitative variables, mean and standard deviation for the quantitative variables with normal distribution, and the median and interquartile range for the quantitative variables that did not have a normal distribution. McNemar’s test was used to analyze the dichotomous variables. After testing for the normality of the continuous variables with the Kolmogorov–Smirnov test, the paired Student’s *t*-test was used to analyze the normally distributed variables and the Wilcoxon signed-rank test was used to analyze the non-parametric variables. A *p*-value < 0.05 was considered statistically significant.

## 3. Results

**Figure 1:**
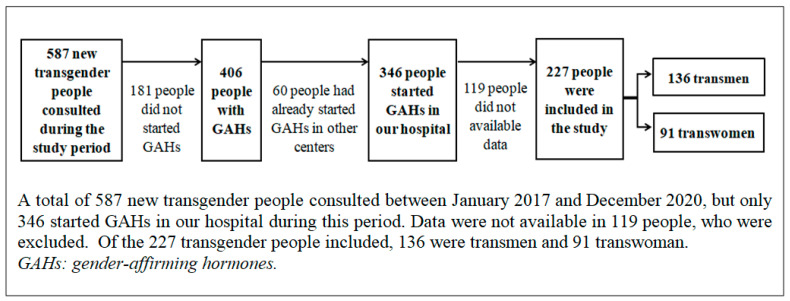
Patient selection procedure and number of patients for each step of the process.

A total of 587 transgender people attended the Multidisciplinary Unit for the Treatment of Sex Reassignment of Cadiz for the first time between January 2017 and December 2020, but only 406 were treated with GAHs. We excluded 60 people who had already started treatment in other centers in the years before and 119 people who had started treatment in our hospital without available data (either due to loss of follow-up or a treatment duration of less than one year at the time of data collection). Out of the 227 transgender people included in the study, 136 (59.91%) were transmen and 91 (40.09%) were transwomen. The selection procedure is shown in Figure 1.

In 2017, a total of 49 patients were recruited (21 transmen and 28 transwomen); in 2018, 49 patients (28 transmen and 21 transwomen); in 2019, 65 patients (47 transmen and 18 transwomen); and in 2020, 64 patients (40 transmen and 24 transwomen). We detected an inversion of the transwoman/transman ratio, which was 1.33 in favor of transwomen during the first year and 1.66 in favor of transmen in the last year. In the second half of the study period, the number of patients who started and completed treatment in our unit increased to 38%.

The transmen started hormonal treatment with a median age of 18 (16–23) years. Only seven (5.1%) transmen were treated with gonadotropin-releasing hormone analogs before GAHs. Regarding surgery, indicated for transgender people over 18 years of age and a minimum year of hormonal treatment, 28 (20.6%) mastectomies, 22 (16.2%) hysterectomy, and 1 (0.7%) masculinizing genitoplasty were performed. The transwomen started GAHs with a median age of 18 (16–22) years. Of these, 10 (11%) had received pubertal suppression. The surgical interventions performed were eight (8.8%) mammoplasties, one (1.1%) Wendler glottoplasty, and eight (8.8%) feminizing genitoplasties.

**Figure 2:**
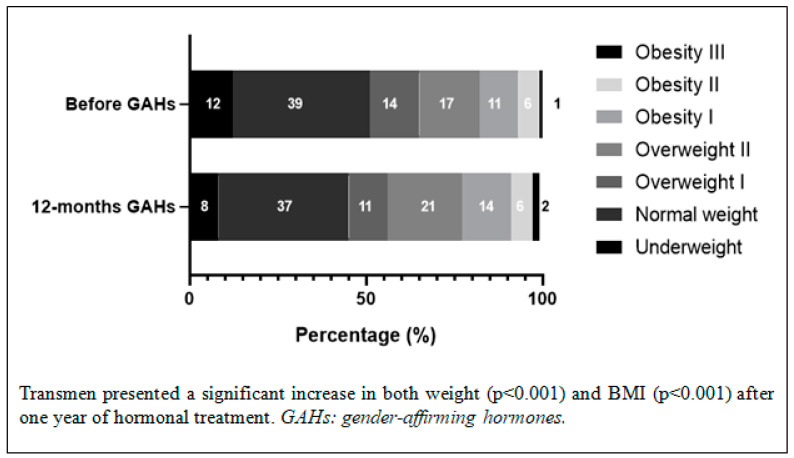
Weight changes in transmen.

**Figure 3:**
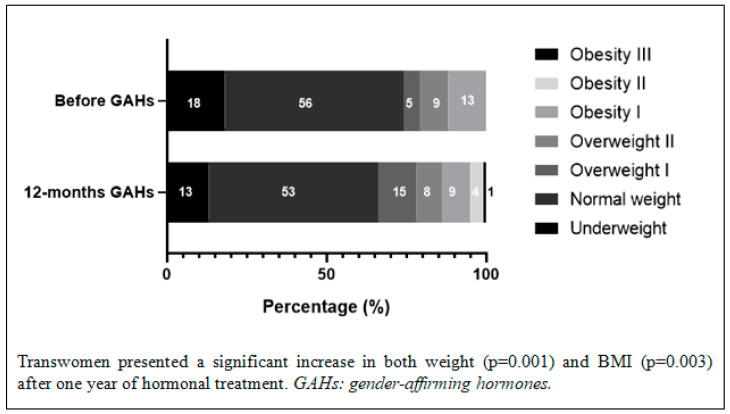
Weight changes in transwomen.

The changes in anthropometric parameters, blood pressure control, and glucose and lipid metabolism are shown in Table 1 and Table 2. The evolution of weight after a year of hormonal treatment is shown in Figure 2 and Figure 3. Both the transmen and the transwomen developed a significant increase in weight, BMI, and systolic and diastolic blood pressure. The transmen showed a worse lipid profile, with a decrease in cholesterol HDL (*p* < 0.001) and an increase in triglycerides (*p* < 0.001) after hormonal treatment, whereas in the transwomen only an increase in total cholesterol (*p* = 0.008) was detected, without relevant changes in the rest of the analyzed parameters. According to the criteria established for dyslipidemia, a significant increase in the development of dyslipidemia (*p* < 0.001 in transmen; *p* = 0.035 in transwoman) was reported. Lifestyle modifications were prioritized in most of the people; only three transmen and one transwoman started treatment with statins.

Regarding the metabolism of carbohydrates, in transmen an increase in glycosylated hemoglobin (*p* = 0.040) was detected, while in transwomen significant changes were detected in fasting blood glucose levels (*p* = 0.008). However, there were no statistically significant changes in the prevalence of prediabetes, and no cases of type 2 diabetes mellitus were reported after treatment. Two transwomen had a diagnosis of type 1 diabetes mellitus: in one of them the insulin requirements remained stable while the other one required a doubling of the total insulin dose (from 50 to 97 UI per day). No thrombotic or major adverse cardiovascular events were reported in either group during the study period.

## 4. Discussion

Since the creation of the Gender Identity Treatment Unit of Cádiz, the request for health care by transgender people has grown exponentially in recent years, although not all patients started GAHs. This trend has also been described in other works such as that of the Amsterdam cohort [20]. Likewise, at the beginning of the study period, we served a higher percentage of transwomen than transmen, but this ratio has changed in recent years. Similar data are reported by observational studies published in other countries in the past decade [20].

The transgender population has a higher prevalence of negative health indicators, including sexually transmitted infections, cardiovascular disease, mental health problems, and substance abuse [21]. However, there are still many areas that have been poorly analyzed and long-term longitudinal follow-up studies are scarce. The human immunodeficiency virus (HIV) is associated with an increased risk of cardiovascular events, which are due to both the direct cardio-metabolic changes of the infection itself and the side effects of antiretroviral treatment. A higher prevalence of HIV infection has been described in transgender people compared to the general population [22]. In our cohort, 1.8% of the subjects were diagnosed with HIV; this proportion is lower than that described by other authors; however, this may be due to the young population we care for, greater health education, and the lack of a systematic determination of HIV serology in our patients during the study period.

Cardiovascular disease is the main cause of morbidity and mortality in the world. Despite this, only 4% of the studies funded by the National Institute of Health for the LGBTQ population between 1989 and 2011 were focused on cardiovascular disease or CVRF. Hence, in 2011 the National Academy of Medicine recommended increasing the research in this field.

Data reported by the Behavioral Risk Factor Surveillance System establish that the transgender population has a higher risk of cardiovascular disease than the general population. Ethinyl estradiol, as part of GAHs in transwomen, has been associated with a higher rate of thromboembolic events [8]; so, the current clinical practice guidelines recommend 17-β-estradiol instead, using transdermal administration to prevent hepatic first-pass metabolism in women over 45 years of age or with a high risk of thrombotic events [6].

In transmen, a higher risk of myocardial infarction has been described compared to ciswomen, which could be related to the changes that the elevation of testosterone induces in arterial intima thickness and atherosclerotic plaque [23,24]. Probably because of the short follow-up of our study and the mainly young population that was treated, we did not detect any cardiovascular or thromboembolic event in either the transmen or transwomen, but changes at the level of the metabolic analytical parameters and the various CVRFs were identified.

Regarding anthropometric parameters, our study confirms a significant increase in weight in transmen and transwomen, although the incidence of overweight/obesity was significantly higher only in transmen. Other authors have described a higher prevalence of obesity in transgender people compared to the general population [15], as well as a significant increase in BMI (from 1.3% to 11.4%) in transmen treated with testosterone in comparison with cisgender women, but not when compared with cisgender men [25]. In transwomen, the most recent studies report similar results to those obtained in our series, but the BMI increase percentages after starting hormonal treatment are variable [26,27,28]. This could be influenced by the anti-androgen drugs used (cyproterone acetate or gonadotropin-releasing hormone analogs in Europe and spironolactone in the US), the method of estrogen administration (oral in Europe and a preference for transdermal delivery in the US), and the duration of hormonal treatment.

It would be interesting to analyze not only the classical anthropometric parameters, but also the changes in the composition of the human body measured by electrical bioimpedance or dual-energy X-ray absorptiometry. A systematic review reported that testosterone therapy increased lean body mass and decreased fat body mass, while transwomen experienced an increase in fat body mass and a decrease in lean body mass [29]. However, studies in this field are few and have small sample sizes.

GAHs induce early sex-specific lipoprotein and apolipoprotein changes in a dose-dependent manner; however, their clinical relevance is still unknown [19]. Reviewing the analytical parameters, these changes are reflected in the appearance of a more atherogenic lipid profile, with maintained LDL cholesterol values and a decrease in HDL cholesterol levels. In transmen treated with testosterone, a significant increase in plasma triglyceride levels was also identified. These results in transmen are consistent with those described by other authors, who report decreases in HDL cholesterol between 3.4% and 23.4% and increases in triglycerides from 7.5% to 44% [30,31].

However, changes in the lipid profile in transwomen were variable in the different series [30,31,32]; this could be influenced by the route of estrogen administration: triglycerides rise more significantly in transwomen treated with oral estradiol than in those treated with transdermal estrogens, a phenomenon that has already been reported in postmenopausal ciswomen. A study even described the favorable effects of estrogen treatment during the first year in transwomen, with a decrease in LDL cholesterol, a reduction in triglycerides, and a slight increase in HDL cholesterol [33].

The study published by Robinson et al. in 2022 explored the interrelationship between sex hormones and lipids in prepuberal children, young post-puberal cisgender people, and transgender individuals on GAHs. In post-puberal ciswomen, a significant increase in HDLc and a decrease in LDLc compared with cismen was reported. These differences were induced by hormonal therapy in transgender people. The atheroprotective profile was related to the upregulation of HDL/APoA1 drives by estradiol [34]. These effects of estrogens explain why women with premature or early menopause (before 45 years old), natural or surgically induced, have more CVRF than women whose ovarian function ceases later.

Regarding the effect of hormonal treatment on glucose metabolism, we detected a significant increase in analytical parameters (fasting blood glucose in transwomen and glycated hemoglobin in transmen), although these changes did not translate into an increased incidence of prediabetes in any group. In a systematic review that evaluated the effect of testosterone on insulin resistance, no negative effects were detected at this level (only two of the thirteen studies showed an increase in insulin resistance) [31]. In contrast, a possible interaction between estrogens and the development of insulin resistance in transwomen has been described, but the results are not conclusive [35]. Neither the Amsterdam cohort [36] nor the Strong cohort [37] have reported differences in baseline prevalence and the incidence of diabetes in transgender people who initiated GAHs compared with reference cisgender male and female controls. However, when examining the incidence of diabetes in transwomen, in both those taking and those not taking GAHs, there was an increased incidence and prevalence of diabetes compared with ciswomen, suggesting the risks of diabetes may not be related to hormonal therapy [38].

Regarding arterial hypertension, our study detected an increase in SBP and DBP in both genders. Several studies report small elevations in SBP, but without clinical significance [39,40]. A more recent study published in 2021, which included a total of 470 subjects, with a follow-up of up to two years, concluded that SBP increased by 2.6 mmHg in transmen and decreased by 4 mmHg in transwomen at four months from the start of GAHs; this was maintained during the follow-up period, whereas the DBP did not change [41]. However, the evidence on the relationship between blood pressure and GAHs may be influenced by various confounding factors, such as age, smoking, and hormonal therapy duration.

The first measure taken in patients with weight gain and non-specific alterations in the metabolic profile was lifestyle intervention, through a nutritional education program taught by nurses. Only four patients required statins after one year of hormonal treatment due to important elevations in LDL cholesterol, according to the clinical practice guidelines for the general population. We did not start metformin in the transgender population with prediabetes; instead, we prioritized physical exercise and dietary advice. In addition, antihypertensive therapy was not prescribed one year after GAHs.

Our study has some limitations. Firstly, its retrospective nature and the short follow-up may not be sufficient to derive definitive conclusions about the association between CVRF and cross-sex hormonal therapy. The follow-up of this cohort will be critical to our understanding of the long-term effects on chronic health and the impact on cardiovascular morbidity and mortality. These results refer to the use of oral estrogens and intramuscular testosterone; so, new studies to confirm whether these changes also occur with transdermal administration would be necessary. It should be noted that the study was carried out at one center, which made it possible to avoid the differences that were secondary to the changes in therapeutic regimens and clinical practices that might exist between centers due to hormone treatment not being entirely standardized.

## 5. Conclusions

The demand for GAHs has grown exponentially in recent years and the treatment is being started at increasingly younger ages. This requires a deep knowledge of the vascular pathophysiology and cardiovascular impact of hormonal therapy, not only of GAHs but also of puberty blockers. The transgender people treated in our hospital showed a tendency to have a worse metabolic profile after starting hormonal treatment; they developed early changes in lipid and carbohydrate metabolism and systolic and diastolic blood pressure control, in addition to a significant gain in weight.

The results suggest the need for lifestyle interventions from the beginning of GAHs to achieve optimal body weight management. It is necessary to raise awareness of the side effects to improve therapeutic adherence to these interventions. Moreover, it would be interesting to discuss targets for CVFR control in the transgender population to develop strategies for primary cardiovascular prevention that allow the taking of early therapeutic actions when necessary. Nowadays, there are no clinical practice guidelines for monitoring CVRF in this group, apart from the recommendations made for the general population. Perhaps, more strict objectives should be established based on the current evidence.

Finally, the results obtained in our study open new lines of research. The use of methods for the assessment of body composition in transgender people would be useful in determining whether the increase in body mass index is related to a gain in muscle mass and/or lean mass. In addition, an analysis of circulating inflammatory cytokines before and after hormonal treatment would contribute to a better understanding of the changes in the metabolic profile reported in our work.

## Figures and Tables

**Table 1:** Anthropometric parameters, analytical determinations and blood pressure control before and one year after GAHs in transmen.

Variable	Before GAHs	A Year after GAHs	*p* Value
Weight (kg) ^1^	66.36 ± 16.52	69.95 ± 18.15	**<0.001**
BMI (kg/m^2^) ^1^	25.31 ± 5.69	26.41 ± 6.14	**<0.001**
FBG (mmol/L)	4.55 (4.14–4.94) ^2^	4.48 ± 0.7 ^1^	0.118
HbA_1c_ (mmol/mol) ^2^	32 (31–33)	33 (32–37)	**0.040**
Total cholesterol (mmol/L)	4.14 (3.68–4.61) ^2^	4.21 ± 0.82 ^1^	0.555
Triglycerides (mmol/L) ^2^	0.8 (0.63–1.18)	0.95 (0.73–1.45)	**<0.001**
LDL–cholesterol (mmol/L) ^2^	2.36 (1.97–2.76)	2.46 (2.07–2.95)	0.134
HDL–cholesterol (mmol/L)	1.37 (1.14–1.58) ^2^	1.23 ± 0.27 ^1^	**<0.001**
SBP (mmHg) ^2^	118.5 (110–127.5)	129 (118–142)	**0.006**
DBP (mmHg) ^2^	71 (64–79)	80.5 (70.5–86)	**0.015**

GAHs = gender-affirming hormones; BMI = Body Mass Index; FBG = fasting blood glucose; HbA_1c_ = glycated hemoglobin_;_ LDL = low-density lipoprotein; HDL = high-density lipoprotein; SBP = systolic blood pressure; DBP = diastolic blood pressure. ^1^ Data expressed as mean ± standard deviation, ^2^ Data expressed as median (interquartile range).

**Table 2:** Anthropometric parameters, analytical determinations and blood pressure control before and one year after GAHs in transwomen.

Variable	Before GAHs	A Year after GAHs	*p* Value
Weight (kg) ^2^	64.5 (56.5–82.1)	67.5 (59.0–82.5)	**0.001**
BMI (kg/m^2^) ^2^	21.63 (19.0–26.9)	22.77 (20.4–26.6)	**0.003**
FBG (mmol/L) ^2^	4.66 (4.33–4.88)	4.77 (4.38–5.11)	**0.008**
HbA_1c_ (mmol/mol) ^2^	32 (30–34)	33 (31–35)	0.552
Total cholesterol (mmol/L)	3.83 (3.3–4.53) ^2^	3.8 ± 0.75 ^1^	**0.008**
Triglycerides (mmol/L) ^2^	0.79 (0.63–1.01)	0.68 (0.58–0.93)	0.115
LDL–cholesterol (mmol/L)	2.28 (1.81–2.94) ^2^	2.26 ± 0.68 ^1^	0.415
HDL–cholesterol (mmol/L)	1.2 (1.01–1.4) ^2^	1.19 ± 0.28 ^1^	0.084
SBP (mmHg)	122 (116.5–133.5) ^2^	126.21 ± 18.78 ^1^	**0.026**
DBP (mmHg) ^2^	74 (67–79)	80.5 (68.5–86.5)	**0.017**

GAHs = gender-affirming hormones; BMI = Body Mass Index; FBG = fasting blood glucose; HbA_1c_ = glycated hemoglobin_;_ LDL = low-density lipoprotein; HDL = high-density lipoprotein; SBP = systolic blood pressure; DBP = diastolic blood pressure. ^1^ Data expressed as mean ± standard deviation, ^2^ Data expressed as median (interquartile range).

## Data Availability

The datasets generated and/or analyzed during the current study are available from the corresponding author upon reasonable request.

## Abbreviations

BMI	body mass index
CVRF	cardiovascular risk factors
DBP	diastolic blood pressure
FBG	fasting blood glucose
GAHs	gender-affirming hormones
HbA_1c_	glycated hemoglobin
LDL	low-density lipoprotein
LGBTQ	lesbian, gay, bisexual, transgender, and queer
HDL	high-density lipoprotein
SBP	systolic blood pressure
HIV	human immunodeficiency virus
